# Next generation sequencing and genomic mapping: towards precision molecular diagnosis of lung cancer in Morocco

**DOI:** 10.11604/pamj.2024.49.75.45306

**Published:** 2024-11-13

**Authors:** Ouafaa Morjani, Noura Mounaji, Meriem Ghaouti, Hassan Errihani, Elmostafa El Fahime, Hamid Lakhiari

**Affiliations:** 1 Laboratory of Virology, Oncology, Biosciences, Environment, and New Energies, Faculty of Sciences and Technics Mohammedia, Hassan II University, Casablanca, Morocco,; 2 Pathology and Molecular Biology Center United Nations, Rabat Morocco,; 3 National Institute of Oncology, Ibn Sina University Hospital Center, Mohammed V University, Rabat, Morocco,; 4 Functional Genomic Platform, National Center of Scientific and Technical Research, Rabat, Morocco,; 5 Mohamed VI Center for Research and Innovation, Mohamed VI University of Health Sciences, Casablanca, Morocco

**Keywords:** Next-generation sequencing, lung cancer, genetic alterations, personalized therapy, driver genes

## Abstract

**Introduction:**

lung cancer is the leading cause of cancer-related deaths worldwide, with a significant incidence in Morocco. The complex epidemiology of this disease in the country necessitates an in-depth analysis of genetic profiles to improve diagnosis and treatment. This study utilizes next-generation sequencing (NGS) to map genetic alterations in Moroccan patients with lung cancer, a field where molecular data is largely lacking. Importantly, this study presents a pioneering analysis of lung cancer in the Moroccan population using next-generation sequencing technology. While previous studies focused on a limited number of genes, our research provides a comprehensive and detailed perspective on the genetic alterations within this cohort, including the generation of an oncoprint.

**Methods:**

this study involved 100 histologically confirmed lung cancer patients. Genetic abnormalities were detected using the NGS technique with the Oncomine Precision Assay GX protocol. Lung biopsy samples were prepared, purified, and sequenced, with the resulting data analyzed to identify significant genetic variants.

**Results:**

the analysis revealed genetic alterations in 13 different genes, with a notable prevalence of mutations in the TP53, KRAS, and Epithelial Growth Factor Receptor (EGFR) genes. TP53 mutations were present in 27% of cases, while KRAS and EGFR showed mutations in 19% and 14% of samples, respectively. Clinically significant mutations were also identified in the ALK, MET, ERBB2, and ROS1 genes, highlighting substantial genomic diversity in this cohort.

**Conclusion:**

the results of this study enhance the understanding of genetic alterations in Moroccan lung cancer patients and underscore the need to strengthen efforts for advanced molecular diagnosis in Morocco. The use of NGS has identified critical genetic mutations, facilitating the development of personalized treatments and improving clinical outcomes. These findings pave the way for future research aimed at refining diagnostic and therapeutic strategies, thereby contributing to better patient management.

## Introduction

Lung cancer, the leading cause of cancer-related deaths worldwide, resulted in approximately 1,796,144 deaths in 2020. In Morocco, the epidemiology of lung cancer is complex and requires a nuanced approach. According to the International Agency for Research on Cancer (IARC) data, in 2020, Morocco recorded 7,353 new cases of lung cancer, including 6,502 men and 851 women. The mortality rate that year was 6,551 deaths, distributed between 5,802 men and 749 women [1]. It is important to note that these figures, although from the IARC, may not fully reflect the current situation. Moroccan lung cancer statistics are primarily derived from two main registers. These registers, such as the Greater Casablanca register (2013-2017) and the Rabat register (2009-2012), have limitations due to limited geographical coverage and a lack of regular updates.

Lung cancer is often diagnosed at an advanced stage, leading to a poor prognosis and necessitating treatments based on various chemotherapy protocols and surgical interventions. Recent advances in genetic sequencing, particularly through NGS and immunotherapy, have enabled the development of personalized treatments aimed at improving overall patient survival. Nucleic acid sequencing began with the Sanger method in the 1970s [2]. Today, advanced NGS technologies allow for billions of simultaneous sequencing reactions. The results are then integrated into online genomic databases to identify and compare specific mutations. NGS can sequence the entire genome in 24 hours, revolutionizing the field of oncology.

Next-generation sequencing (NGS) is of major interest in the treatment of lung cancer due to its ability to quickly and accurately identify patient-specific genetic mutations. This precision allows for personalized treatments, increasing the chances of therapeutic success. By detecting target mutations, NGS facilitates the application of targeted therapies and immunotherapies, significantly improving survival rates and the quality of life for patients. Additionally, NGS plays a crucial role in monitoring cancer progression and adjusting treatments in real-time. This study aims to fill the current gaps in lung cancer data in Morocco, focusing on genetic mutations detectable by NGS technologies. The specific objectives of this study are to identify frequent genetic alterations in Moroccan lung cancer patients, compare these findings with those reported in other populations, and propose personalized therapeutic approaches based on the observed genetic profiles. By providing a detailed mapping of mutations, this research aspires to improve the diagnosis, treatment, and clinical management of lung cancer patients in Morocco. As the first study to utilize NGS and generate an oncoprint for Moroccan lung cancer patients, it sets a significant benchmark for future molecular research in the region.

## Methods

**Study design:** this study is a molecular analysis using next-generation sequencing (NGS) involving patients with histologically confirmed lung cancer diagnosed by expert pathologists. The primary objective was to identify genetic abnormalities associated with this disease in the Moroccan population using the Oncomine Precision Assay GX protocol with NGS technology.

**Setting:** the study was conducted at the United Nations Laboratory of Anatomical Pathology and Molecular Biology in Rabat, Morocco.

**Participants:** a total of 100 patients with histologically confirmed lung cancer were included in the study. Lung biopsy samples were fixed in buffered formalin and embedded in paraffin.

**Variables:** the genetic abnormalities detected in the study include mutations, amplifications, and other genetic alterations, in addition to the clinical parameters of the patients.

**Data sources/measurement:** genetic abnormalities were detected using the NGS technique with the Oncomine Precision Assay GX protocol. Samples were prepared by cutting 10-micrometer sections, deparaffinized using xylene, and rehydrated through a series of ethanol washes. Proteinase K digestion was then performed to release high-quality nucleic acids, which were purified using specific columns or magnetic beads to remove impurities. Nucleic acids were quantified using Qubit kits, and their integrity was assessed with the Agilent 2100 Bioanalyzer. Library preparation involved the controlled fragmentation of DNA and RNA, end repair, and ligation of adapters containing barcodes for sample identification. After amplification, the libraries were loaded onto a GX5™ chip for sequencing on the integrated Genexus sequencer using Ion Torrent technology. The sequencing process detects hydrogen ions released during nucleotide incorporation, converting these signals into readable sequences. The data were analyzed using Ion Torrent™ Suite software, aligning the reads to the human reference genome (GRCh38), identifying genetic variants, and annotating them with clinical databases such as ClinVar and COSMIC.

**Bias:** since the study is based on available and confirmed samples, there may be selection bias related to the quality and quantity of genetic material in the samples. This could limit the generalizability of the findings to the broader Moroccan population.

**Study size:** a total of 100 patients were included in this study, providing sufficient statistical power to identify frequent mutations in the genes studied.

**Quantitative variables:** the genetic mutations observed in each gene were quantified by their allelic frequency, determined from the sequencing results. The age distribution, gender, and tumor histology of the patients were also analyzed as quantitative variables.

**Statistical methods:** the sequencing results were analyzed using descriptive statistical tests to identify the frequencies of mutations in the genes of interest. The correlation between genetic mutations and the clinical characteristics of the patients (age, gender, histological type) was evaluated using Chi-square tests or Fisher's exact tests, where applicable. A significance threshold of p<0.05 was used for all statistical tests.

## Results

**Figure 1:**
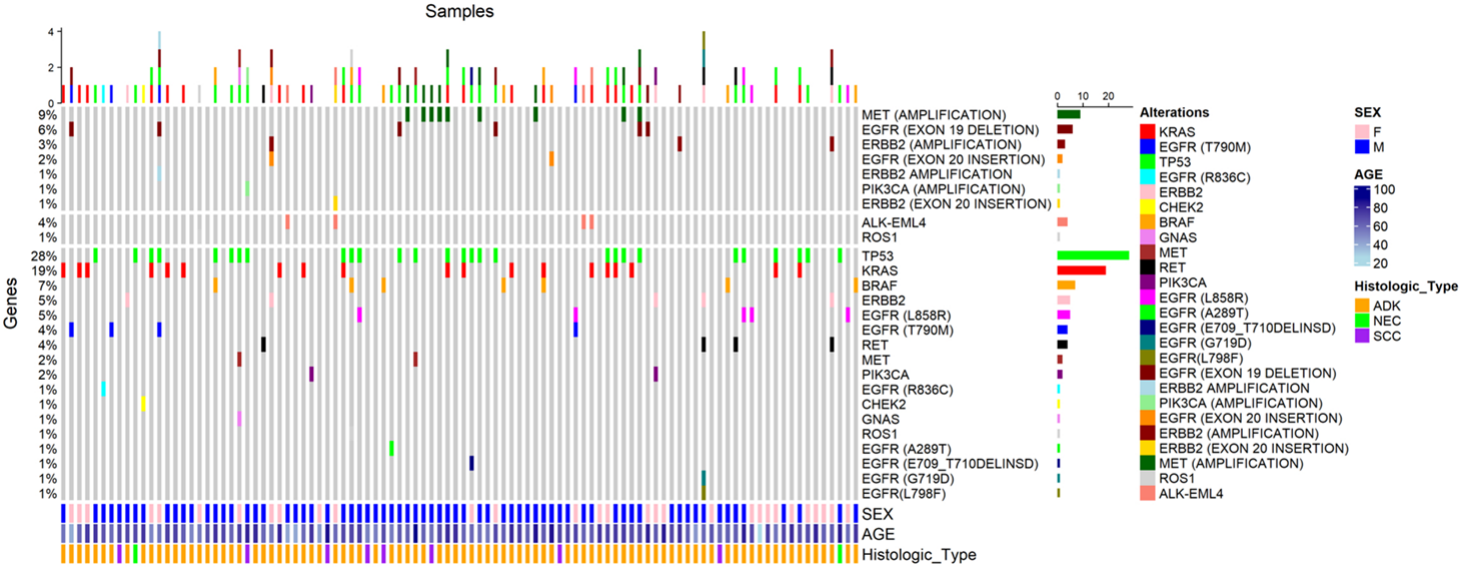
oncoprint representing genetic alterations in lung cancer patients, different colors indicate various types of mutations, while the bars represent the frequency of these alterations in the samples

**Descriptive data:** the distribution of genetic mutations observed in our cohort of lung cancer patients is detailed through the oncoprint presented below, generated with R (Figure 1). This oncoprint provides a clear and concise visualization of the various genetic alterations identified in the studied samples. Each column in the oncoprint corresponds to a patient sample, while each row represents a specific gene. Different colors indicate the various types of genetic mutations, such as amplifications, deletions, and insertions, as well as point mutations and gene fusions. This figure highlights the frequency and diversity of genetic alterations in our cohort, illustrating the most frequently mutated genes, such as TP53, KRAS, and EGFR, and showing the different combinations of mutations present in patients. The oncoprint also allows for the visualization of the correlation between genetic mutations and the clinical and demographic characteristics of patients, such as gender, age, and histological type of cancer.

**Participants:** the gender distribution among lung cancer patients shows a male predominance, with 70 men (70%) versus 30 women (30%), resulting in a sex ratio of 2.33 (Table 1). A wide variety was observed in the age ranges. The minimum age is 26 years, while the maximum age is 90 years. The average age is 65.37 years, and the median age is 67 years. The age range with the highest number of patients is between 60 and 70 years.

**Table 1:** summary of baseline patient characteristics

Patient characteristics	N=100	%
**Gender**		
Female	30	30%
Male	70	70%
**Age, years**		
Minimum	26	N/A
Maximum	90	N/A
Mean	65.37	N/A
Median	67	N/A
**Histologic types**		
Adenocarcinoma	90	90%
Squamous cell carcinoma	8	8%
Neuroendocrine carcinoma	2	2%
N/A (not applicable)	4	4%

**Figure 2:**
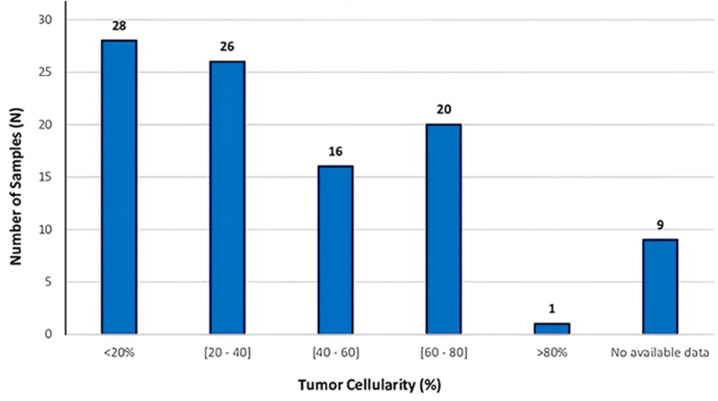
distribution of tumor cellularity across different ranges

**Outcome data:** the collected data show that the majority of analyzed samples come from solid biopsies, with a total of 86 samples. Liquid biopsies are less common, representing only 7 samples. We also examined 4 samples from lobectomy resection specimens and 2 samples from pulmonary wedge resections. Finally, centrifuged pleural fluid pellets are the rarest, with only one sample identified. The results of the tumor cellularity analysis show a varied distribution among the samples. The majority of samples have a tumor cellularity of less than 20%, with 28 samples in this category. A similar number of samples, 26, have a tumor cellularity between 20% and 40%. The range of tumor cellularity between 40% and 60% includes 16 samples, while 20 samples have a tumor cellularity between 60% and 80%. Only one sample has a tumor cellularity greater than 80%. Additionally, no tumor cellularity data are available for 9 samples (Figure 2).

**Main results:** the study revealed genetic abnormalities in 13 different genes, with a predominance of mutations in the TP53 gene, which has a total of 27 distinct mutations (accounting for 27% of the total number of cases), with 77.78% of the cases being men and 22.22% being women. Cases with mutated TP53 were predominantly over 60 years old (70.37%) and diagnosed with the ADK histological type (85.18%). However, 2 cases were classified as squamous cell carcinomas (7.40%), and 2 other cases as neuroendocrine carcinomas (7.40%). The mutations detected in TP53 include: G245S (c.733G>A; AF=3.19%), P151R (c.452C>G; AF=14.18%), p.C176F (AF=0.196%), E285K (c.853G>A; AF=6.12%), K132M (c.395A>T; AF=49.63%), Y236D (c.706T>G; AF=0.25%), V157F (c.469G>T; AF=22.53%), R249S (c.747G>T; AF=23.72%), R175H (c.524G>A; AF=0.15%), C135Y (c.404G>A; AF=44.88%), R248Q (c.743G>A; AF=3.27%), R156P (c.467G>C; AF=11.66%), V216M (c.646G>A; AF=37.48%), C242F (c.725G>T; AF=11.43%), C176W (c.529_546delCCCCACCATGA GCGCTGC; AF=8.26), C176G (c.528C>G; AF=6.19%), C177-P182del (c.526T>G; AF=6.44%), V272L (c.814G>T; AF=60.37%), c.832C>A (AF=0.102%), G244S (c.730G>A; AF=18.74%), R273G (c.817C>G; AF=45.80%), G262V (C785G>T; AF=3.61%), D281G (c.842A>G; AF=26.91%), M237L (c.709A>T; AF=4.19%), G154V (c.461G>T; AF=48.82%), E286Q (c.856G>C; AF=30.52%), and Q192* (c.574C>T; AF=44.67%).

Notably, the mutations C176W (c.529_546delCCCCACCATGAGCGCTGC; AF=8.26), C176G (c.528C>G; AF=6.19%), and C177-P182del (c.526T>G; AF=6.44%) were found simultaneously in the same patient. Additionally, the R175H mutation was observed in three different patients, representing 11.11% of cases with mutated TP53 (Table 2). The other mutations were less frequent, each representing one case.

**Table 2:** allelic frequency distribution of the TP53 R175H mutation across different cases

Gene	Mutation	Cases	Codons	Allele frequency
TP53	R175H	Case 1	c.818G>A	0.93%
			C815T>A	1.17%
			c.814G>A	0.39%
			c.721T>C	0.13%
			c.700T>C	0.16%
			C.659A>G	0.97%
			c.578A>G	0.34%
			c.535C>A	0.14%
			c.524G>A	1.34%
			c.434T>C	0.14%
		Case 2	c.524G>A	16.86%
		Case 3	c.524G>A	0.15%

**Figure 3:**
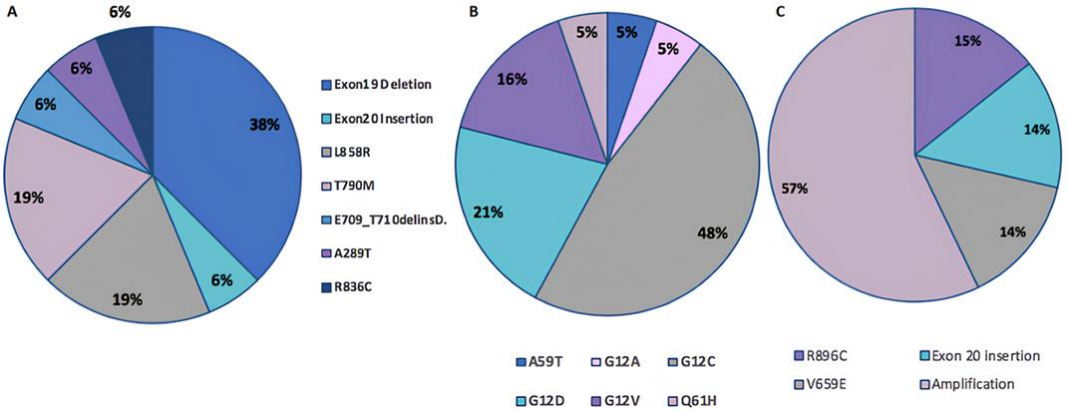
distribution of genetic alterations in study samples: A) frequency of exon alterations in the EGFR gene; B) distribution of KRAS alterations; C) distribution of alterations and amplifications in the ERBB2 gene

The EGFR gene was found to be mutated in 14% of cases (Figure 3A). This incidence was evenly distributed between men and women, each representing 7% of the mutated cases, and primarily involved patients over 60 years old. Among the identified mutations, exon 19 deletion was the most frequent, accounting for 38% of the observed mutations (Table 3). The L858R mutations (c.2573T>G; AF=69.12%), (c.2573T>G; AF=23.32%), (p.L858R; AF=0.14%) and T790M mutations (c.2369C>T; AF=15.13%), (c.2369C>T; AF=0.91%), (c.2369C>T; AF=26.38%), were also frequent, each representing 19% of the samples. Lastly, insertions at exon 20 (c.2311_2312insGCGTGGACA; AF=27.89%), and the mutations R836C (c.2506C>T; AF=18.10%), A289T (c.865G>A; AF=2.76%) and E709_T710delinsD (c.2127_2129delAAC; AF=0.65%) were each present in 6% of the samples.

**Table 3:** summary of EGFR Exon 19 deletions

Gene	Mutation	Codons	Allele frequency
EGFR	EGFR19del	c.2238_2248delATTAAGAGAAGinsGC	67.43%
		c.2237_2255delAATTAAGAGAAGCAACATCinsT	49.48%
		c.2235_2249delGGAATTAAGAGAAGC	10.43%
		c2235_2249delGGAATTAAGAGAAGC	82.89%
		c.2236_2253delGAATTAAGAGAAGCAACA	37.28%
		c.2235_2249delGGAATTAAGAGAAGC	5.68%

The KRAS gene is mutated in 19% of cases or 19 out of 100 patients. Among these 19 cases, 13 involve men (68%) and 6 involve women (32%). Among the identified mutations, the G12C mutation (c.34G>T; AF=4.82%), (c.34G>T; AF=6.88%), (c.34G>T; AF=58.48%), (c.34G>T; AF=38.97%), (c.34G>T; AF=23.21%), (c.34G>T; AF=35%), (c.34G>T; AF=46.01%), (c.34G>T; AF=36.31%), (c.34G>T; AF=3%) was the most frequent, representing 48% of the observed mutations (9 out of 19 cases). This mutation is followed by the G12D mutations (c.35G>A; AF=47.23%), (c.35G>A; AF=58.19%), (c.35G>A; AF=6%), (c.35G>A; AF=51.17%) and G12V mutations (p.G12V; AF=0.261), (c.35G>T; AF=19.71), (p.G12V; AF=0.137%), representing 21% (4 cases) and 16% (3 cases) of the samples, respectively. The A59T (c.175G>A; AF=0.12%), G12A (c.183A>C; AF=12.85%), and Q61H (c.35G>C; AF=3.32%) mutations were less frequent, each present in 5% of the samples (1 case each) (Figure 3B).

Regarding the ERBB2 gene, it was found to be mutated in 7% of the cases studied in our cohort, evenly distributed between 4 men and 3 women. The identified mutations include R896C (c.2686C>T; AF=46.50%) with 1 occurrence, an insertion in exon 20 (c.2340_2341insGGCTCCCCA; AF=9.82%) also with 1 occurrence, V659E (c.1976_1977delTTinsAG; AF=64.73%) with 1 occurrence, and finally, ERBB2 gene amplification, which is the most frequent with 4 occurrences, with copy numbers varying (copy number: 14 - 5.8 - 21.18 - 5.4) (Figure 3C). BRAF gene mutations were identified in 7% of cases, all in men over the age of 60. The V600E mutation (c.1799T>A; AF=13.39%), (AF=26.89%) was detected in two cases, followed by the A598T (c.1792G>A; AF=0.08%), G466V (c.1397G>T; AF=25.04%), G596R (c.782C>T; AF=19.89%), K601E (c.1801A>G; AF=8.85%) and V600-K601delinsE (c.1799_1801delTGA; AF=28.51%) mutations, each present in one case. The V600-K601delinsE mutation involves the deletion of valine (V) at position 600 and lysine (K) at position 601, replaced by an insertion of a glutamate (E) amino acid. We identified ALK gene fusions in 5% of cases, corresponding to a total of five patients. Among these, two were women and three were men. Two of these cases involved patients under the age of 50. The EML4 gene was found to be the fusion partner of ALK in 80% of the observed cases (Table 4). However, one particular case among our observations presented a genetic imbalance at the ALK gene precisely at position chr2: 29455169 (ALK imbalance). Additionally, a mutation at position chr2: 29416366 with an allele frequency of 44.92% (AF=44.92%), characterized by a nucleotide substitution in the complementary DNA, c.4587C>G, resulting in an amino acid change from aspartate to glutamate at position 1529 of the ALK protein (p.(D1529E)).

**Table 4:** summary of EML4-ALK and ALK genetic variants

Genes	Sex	Age	Locus	Variant ID
EML4-ALK	M	61	chr2:42491871-chr2:29446412	EML4-ALK-E6 ins18A20.1.
			chr2:42492091-chr2:29446394	EML4-ALK.E6bA20.AB374362.1
	F	26	chr2:42491871-chr2:29446412	EML4-ALK-E6 ins18A20.1.
			chr2:42492091-chr2:29446394	EML4-ALK.E6bA20.AB374362.1
	M	69	chr2:42552694-chr2:29446394	EML4-ALK-E20A20.COSF409.2
	M	40	chr2:42543190-chr2:29446394	EML4-ALK.E18A20.COSF487.1
ALK	F	58	chr2:29455169	-

MET gene anomalies were detected in 8% of the samples studied. Among these anomalies, 75% were gene amplifications with the following copy numbers: 31.60; 7.00; 4.30; 4.08; 4.05; 4.00. A MET gene fusion was observed in one sample, involving exon 14 skipping. The variant identifier is MET-MET.M13M15.1, located on chromosome 7 at positions chr7: 116411708 - chr7: 116414935. Additionally, the COSM6917314 variant, associated with the NM_001127500.3 transcript (p.D10284), was identified in the same case presenting the MET-MET.M13M15.1 fusion, at position chr7: 116412043 (c.3082G>T) with an allele frequency of 12.73%. This variant has a missense effect. Another missense mutation, p.R988C (c.2962C>T), was noted in the MET gene, located at position chr7: 116411923, with an allele frequency of 0.07%. Various anomalies in the RET gene were observed, representing 3% of all detected mutations. A C618* mutation was identified at position chr10: 43609098 (c.1854C>A) with an allele frequency of 16.85%. A second mutation, R912W, was detected at position chr10: 43617397 (c.2734C>T) with an allele frequency of 3.09%. Finally, the R912Q mutation was identified at position chr10: 43617398 (c.2735G>A) with an allele frequency of 3.81%.

The PIK3CA gene was found to be mutated in three patient samples, involving two men and one woman. The detected mutations include p.H1047R (c.3140A>G) located at position chr3: 178952085, with an allele frequency of 31.28%. The p.E545K mutation (c.1633G>A) was located at position chr3: 178936091, with an allele frequency of 15.95%. Finally, the p.E542K mutation (c.1624G>A) was identified at position chr3: 17893682, with an allele frequency of 4.31%.

Anomalies in the ROS1 gene were detected in 2% of the analyzed samples. Among these anomalies, a specific fusion involving the CD74 and ROS1 genes was identified. This fusion, CD74-ROS1, associates exon 6 of the CD74 gene with exon 34 of the ROS1 gene. The genomic position of this fusion is located on chromosome 5 at position 149784243 and on chromosome 6 at position 117645578 (chr5: 149784243 - chr6: 117645578).

The GNAS and CHEK2 genes were each found to be mutated in 1% of the analyzed cases. Among the detected mutations, the p.R201H mutation in the GNAS gene (c.602G>A) was identified at position chr20: 57484421, with an allele frequency (AF) of 62%. This mutation is of the missense type. Additionally, a p.R523H mutation in the CHEK2 gene (c.1568G>A) was located at position chr22: 29083949, with an allele frequency of 11%. This mutation is also of the missense type.

**Figure 4:**
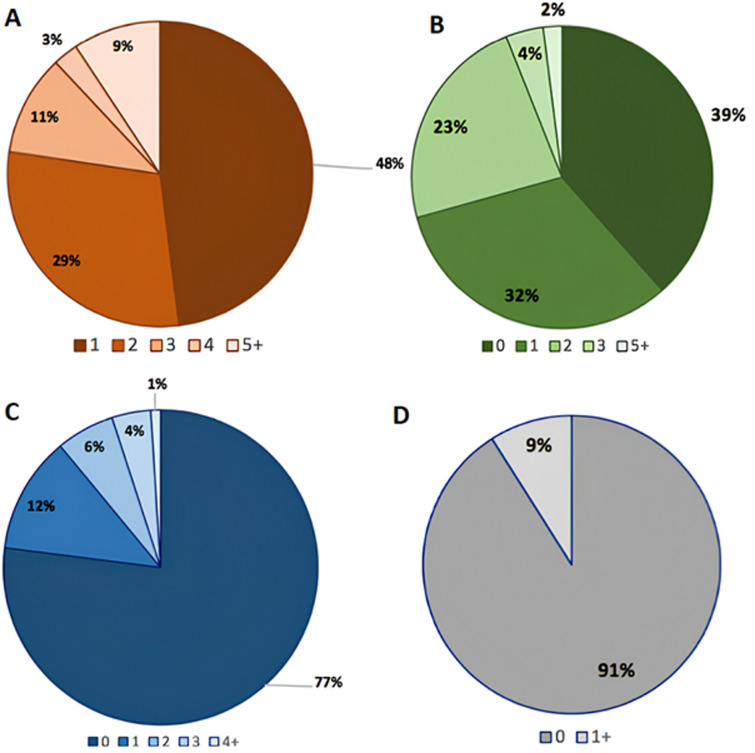
frequency of patients with; A) one or more concomitant alterations, considering all types of gene alterations; B) one or more gene variants; C) one or more copy number alterations (CNAs); D) one or more rearrangements

The study results show that concomitant alterations, variants, CNAs, and rearrangements are distributed variably, with notable proportions of samples presenting one or more alterations in each category (Figure 4).

## Discussion

The molecular profile of lung cancer in Morocco remains incomplete due to several financial and technical challenges. NGS, while essential for these analyses, is expensive and requires advanced infrastructure that few Moroccan laboratories can maintain due to budget constraints. Limited access to modern equipment and a lack of adequate technical support make it difficult to perform complex tests. Additionally, the unequal distribution of healthcare services across the country hinders the collection of comprehensive and representative data, affecting a thorough understanding of the molecular profile of lung cancer. The absence of national policies promoting genetic testing also limits the development of exhaustive databases. Moreover, many laboratories lack the high-tech equipment necessary for NGS tests, creating additional difficulties for conducting sophisticated molecular analyses. Finally, the lack of integration of clinical research into healthcare impedes the systematic collection of molecular data, essential for guiding treatments and improving patient outcomes. These cumulative obstacles make it challenging to establish a complete and reliable molecular profile of lung cancer in Morocco.

To address these gaps, this study was conducted using NGS technology on biopsies from a cohort of Moroccan lung cancer patients. This technique allows for precise and sensitive detection of mutations, even at low allele frequencies, and offers comprehensive detection capabilities, including various genetic alterations such as substitutions, insertions, deletions, rearrangements, and copy number variations. NGS enables the simultaneous analysis of multiple genomic regions, unlike serial sequencing which targets fewer regions, takes more time, and exhausts biological samples.

The objective of this study is to detect various genetic anomalies within the DNA of these patients, allowing for a better understanding of the disease's genetics, comparison with previous studies in the literature, and guidance toward personalized treatment alternatives and targeted therapies. Lung cancer generally shows significant differences between sexes. Indeed, men have higher tobacco consumption and more frequent exposure to occupational carcinogens, such as asbestos and industrial chemicals. Biological differences also play a crucial role; men and women have different DNA repair mechanisms and immune responses, influencing their susceptibility to lung cancer [3]. Additionally, sex hormones, such as estrogens in women, may exert protective effects by modulating the pulmonary inflammatory and immune response, a mechanism less pronounced in men [4]. Our study aligns with this, noting a male predominance with a sex ratio of 2.33.

The results of our study show a predominance of solid biopsies, with 86 samples, followed by liquid biopsies with 7 samples. Lobectomy resection specimens, pleural fluid centrifugation pellets, and pulmonary wedge resections are less frequent, representing 4, 1, and 2 samples, respectively. Liquid biopsies, although minimally invasive and suitable for continuous disease monitoring, present technical challenges. Their sensitivity may be lower than that of solid biopsies, and the isolation and analysis of circulating tumor DNA can compromise result accuracy. Conversely, solid biopsies, although invasive and sometimes difficult to perform due to access to certain tumors, offer significant advantages. They allow for in-depth genetic mutation analysis and precise tumor localization, essential for comprehensive histopathological evaluation [5]. These characteristics justify their predominant use in our study.

Tumor cellularity varies widely among samples, posing significant challenges for genetic analysis. Low cellularity, observed in a significant proportion of samples (less than 20%), can complicate the extraction and analysis of tumor DNA. The accuracy and reliability of genetic test results largely depend on the amount of available tumor material [6]. However, tumor DNA was effectively extracted using optimized protocols for low-cellularity samples. This allowed successful NGS implementation, ensuring detailed and precise analysis of present genetic mutations. The adoption of advanced sequencing techniques, coupled with improved purification methods, overcame obstacles posed by low tumor material, ensuring the reliability and robustness of the obtained results. Genetic mutations observed in the TP53, EGFR, and KRAS genes are particularly frequent among the analyzed samples, with percentages of 27%, 14%, and 19%, respectively.

Mutations in the TP53 gene play a fundamental role in lung carcinogenesis by altering the function of the p53 protein, often referred to as the “guardian of the genome”. These mutations lead to a loss of p53's ability to regulate the cell cycle and induce apoptosis in response to DNA damage, thus allowing uncontrolled proliferation of damaged cells. Monti *et al*. [7] demonstrated that this loss of p53 function due to TP53 mutations affects various cellular processes crucial for maintaining genomic stability. Furthermore, Barta *et al*. [8] identified that specific TP53 mutations in lung cancer can confer oncogenic gain-of-function, further enhancing tumor progression. TP53 mutations in lung cancer show significant variations across ethnic groups. Among African Americans, these mutations are associated with increased risk and poorer prognosis compared to Caucasians, with a notable prevalence of disruptive mutations [9,10]. In Caucasians, the frequency of TP53 mutations is particularly high in squamous cell carcinomas, reaching 65% [11]. Asian patients, especially non-smokers, exhibit a higher prevalence of TP53 mutations compared to other ethnic groups [12]. Specific data on North African and African populations are limited, but it is established that patients of African descent show a high frequency of disruptive TP53 mutations, correlated with poor prognosis [13]. For Moroccan patients, no prior study has been conducted regarding TP53 mutations in lung cancer.

Among the most frequently identified mutations in the TP53 gene, the R175H (c.524G>A), R273G (c.817C>G), and V157F (c.469G>T) mutations are particularly notable for their association with loss of cell cycle regulation and apoptosis induction, leading to uncontrolled cell proliferation [7,8]. Additionally, the R248Q (c.743G>A) and G245S (c.733G>A) mutations, frequently observed in smoking-related cancers, affect codons essential for p53 stabilization. The V216M (c.646G>A) mutation also deserves particular attention due to its significant impact on p53's suppressive function, contributing to alterations in tumor suppression mechanisms [14]. EGFR gene mutations affect cellular signaling pathways, leading to uncontrolled cell proliferation and suppression of apoptosis. These mutations are particularly frequent in lung adenocarcinomas, especially in non-smoking women and patients of Asian origin [15]. In the Moroccan population, the rate of these mutations remains lower than that of Asians but higher than that of the Caucasian population [16-18].

In our study, the EGFR gene was found to be mutated in 14% of cases (Figure 3A). This incidence was evenly distributed between men and women, each representing 7% of the mutated cases. Among the identified mutations, exon 19 deletion was the most frequent, accounting for 38% of the observed mutations (Table 3). This incidence was slightly lower than that described in other studies involving Moroccan patients [16-18]. This difference may be explained by the variation in population sample characteristics. Additionally, detection methodologies used may differ, influencing detection rates. Patient inclusion criteria, distinct genetic and environmental factors, and sample size may also contribute to this divergence. EGFR gene mutations are crucial in lung carcinogenesis and strongly influence therapeutic choices. Tyrosine kinase inhibitors (TKIs) like gefitinib and erlotinib target mutated EGFR receptors, inhibiting cellular signaling and uncontrolled proliferation [19]. Activating mutations in exon 19 and L858R are major targets of these treatments [20]. A major challenge is the development of resistance to TKIs, particularly the secondary T790M mutation, which confers resistance to first- and second-generation inhibitors [21]. Osimertinib, a third-generation inhibitor, has shown efficacy against tumors harboring this mutation [22]. Additionally, the activation of error-prone DNA replication pathways, induced by GAS6 and AXL, may contribute to therapeutic resistance [23].

In our study, KRAS gene mutations are present in 19% of the cases studied, with G12C being the most frequent (48%), followed by G12D (21%), G12V (16%), and A59T, G12A, Q61H (5% each). In the study by Dumenil *et al*. [24] involving a cohort of 635 NSCLC patients, 19.35% had KRAS gene mutations, a rate remarkably similar to that observed in our study. These mutations were more frequent in male patients, smokers, and histologically diagnosed with adenocarcinoma. Codons 12 and 13 represent the regions most frequently affected by KRAS gene mutations, with G12C being the most common [25,26]. These results are in agreement with those of our study.

Current therapeutic options for KRAS mutations, particularly KRAS G12C, include specific inhibitors like sotorasib and adagrasib, which have shown significant benefits in patients with KRAS G12C-mutant NSCLC [27]. Next-generation inhibitors like RMC-6291 outperform previous ones in preclinical models [28]. Combinatorial strategies, such as SHP2 or RTK inhibition, enhance efficacy by overcoming adaptive reactivation of signaling pathways [29]. Efforts are also underway to develop specific inhibitors for other KRAS mutations, such as G12D and G12V, as well as pan-KRAS agents for a more comprehensive therapy [30].

In addition to TP53, KRAS, and EGFR gene mutations, our study identified genetic alterations in several other genes, highlighting the diversity and genomic complexity of lung cancer in our patient cohort. The ERBB2 gene presented mutations in 7% of cases, mainly including the R896C mutation and an insertion in exon 20. These results are consistent with those of Pasau *et al*. [31], who observed a similar prevalence of ERBB2 mutations in lung adenocarcinomas, and those of Yu *et al*. [32], who identified mutations in the V659/G660 transmembrane domain with much lower frequencies of 0.14% in adenocarcinomas and 0.09% in all lung cancers. BRAF gene mutations were identified in 7% of patients, with a predominance of the V600E mutation. This observation is consistent with the work of Wang *et al*. [33], who also found a similar frequency of BRAF mutations in lung cancers. However, a study by Senior *et al*. [34] reported a BRAF mutation frequency of only 4% in non-small cell lung tumors.

ALK gene fusions, detected in 5% of samples, primarily combined with the EML4 gene, highlighting the importance of these alterations for targeted therapy options, as demonstrated by the study of Rogers *et al*. [35]. In contrast, a study by Harlé *et al*. [36] reported an ALK fusion frequency of only 2.1% in a similar cohort of Caucasian patients. MET gene anomalies, present in 8% of samples, mainly included gene amplifications and a fusion involving exon 14 skippings, consistent with the findings of Babey *et al*. [37]. However, Osoegawa *et al*. [38] reported a high frequency of LKB1 mutations in other subtypes of lung carcinomas, suggesting variability in MET mutations according to histological subtypes.

We observed several anomalies in the RET gene, representing 3% of all detected mutations, including the C618* mutation (chr10: 43609098, c.1854C>A) with an allele frequency of 16.85%, R912W (chr10: 43617397, c.2734C>T) with an allele frequency of 3.09%, and R912Q (chr10: 43617398, c.2735G>A) with an allele frequency of 3.81%. Studies have explored these mutations in the context of lung cancer, showing significant results. The C618* mutation has been associated with translocations and amplifications of the RET gene in lung adenocarcinomas, characterized by a low frequency of translocation but a high incidence of copy gains and amplifications [39]. The R912W mutation, studied in lung cancers, revealed RET gene rearrangements, particularly in adenocarcinoma patients with minimal tobacco exposure. These mutations, although rare in small-cell lung cancers, lead to increased cell proliferation and activation of mitogenic pathways. Additionally, the R912Q mutation has been identified in cases of early and intermediate-stage non-small cell lung cancer, highlighting the importance of targeting these mutations for potential treatment [40].

The results obtained in our study reveal three distinct mutations in the PIK3CA gene in samples from three patients, involving two men and one woman. The detected mutations include p.H1047R (c.3140A>G) with an allele frequency of 31.28%, p.E545K (c.1633G>A) with an allele frequency of 15.95%, and p.E542K (c.1624G>A) with an allele frequency of 4.31%. These observations align with recent literature results. Daher *et al*. [41] reported a 4.1% incidence of PIK3CA mutations in a cohort of 1,377 advanced NSCLC patients, corresponding to the presence of these mutations in our samples. Sestokaite *et al*. [42] detected PIK3CA mutations in 29.3% of NSCLC cases through circulating tumor DNA monitoring, indicating a notable incidence that reinforces the relevance of our results. Additionally, Li *et al*. [43] found PIK3CA mutations primarily in patients under 60 years old, suggesting age-related variation, although our sample is limited to draw similar conclusions. Finally, Liu *et al*. [44] showed that PIK3CA mutations can emerge as resistance mechanisms to EGFR-TKIs, highlighting the clinical challenges posed by these mutations in targeted lung cancer therapy.

Recent research on the ROS1 gene in NSCLC corroborates our observations. In our study, a 2% incidence of ROS1 gene anomalies was detected, with a specific fusion involving the CD74 and ROS1 genes, associating exon 6 of the CD74 gene with exon 34 of the ROS1 gene. This fusion, CD74-ROS1, is located on chromosome 5 at position 149784243 and on chromosome 6 at position 117645578. These results are consistent with those of Zhou *et al*. [45], who found a 0.5% ROS1 rearrangement frequency in Qujing patients, compared to 2.02% in other Yunnan regions. Additionally, Muminovic *et al*. [46] reported ROS1 fusions in 1%-2% of NSCLC cases, aligning with our 2% incidence. Finally, Nadal *et al*. [47] demonstrated the high efficacy of crizotinib in patients with ROS1 gene fusions, highlighting the clinical importance of these rearrangements. These results reinforce the relevance of our observations and highlight the importance of ROS1 gene rearrangements in NSCLC. Recent studies on GNAS gene mutations in non-small cell lung cancer (NSCLC) highlight important findings, although the specific incidence is not always clearly defined. A study using NGS techniques identified GNAS gene mutations in NSCLC patients, without specifying their exact incidence [48]. Another case study showed that trametinib treatment was promising in a patient with osimertinib resistance harboring GNAS R201C and R201H mutations, suggesting a potential treatment option for patients with these activating mutations [49]. Additionally, a study indicated that the YVMA insertion in GNAS mutations is associated with a high incidence of brain metastases and poorer chemotherapy outcomes in advanced NSCLC patients with HER2 kinase domain mutations [50]. Our results show that the GNAS and CHEK2 genes were each found to be mutated in 1% of the analyzed cases. Among the detected mutations, the p.R201H mutation in the GNAS gene (c.602G>A) was identified at position chr20: 57484421, with an allele frequency (AF) of 62%. This mutation is of the missense type. Additionally, a p.R523H mutation in the CHEK2 gene (c.1568G>A) was located at position chr22: 29083949, with an allele frequency of 11%. This mutation is also of the missense type. These results corroborate recent study observations, highlighting the clinical importance of GNAS mutations in NSCLC, particularly in terms of treatment resistance and metastases.

The analysis of genetic alterations in Moroccan lung cancer patients revealed a significant prevalence of mutations in the TP53, KRAS, and EGFR genes, reflecting trends observed in other populations but with particularities specific to our cohort. TP53 mutations, present in 27% of cases, underscore the crucial role of this gene as the guardian of the genome and its impact on cell cycle regulation and apoptosis. KRAS gene alterations, identified in 19% of samples, and EGFR gene alterations, detected in 14% of cases, demonstrate the importance of cellular signaling pathways in lung carcinogenesis.

Additionally, our study highlighted less frequent but clinically significant mutations in the ALK, MET, ERBB2, and ROS1 genes, among others. These findings indicate genomic diversity that could influence responses to targeted therapies and immunotherapies, emphasizing the importance of precise genetic characterization for the development of personalized therapeutic strategies. It is crucial to strengthen initiatives aimed at improving the molecular diagnosis of lung cancer and other types of cancer in Morocco. A detailed analysis of the genetic profiles specific to Moroccan patients will allow for the development of more precise and individualized treatments, thus optimizing clinical outcomes. Moreover, advanced molecular diagnosis enables the early detection of genetic mutations, paving the way for early and potentially more effective therapeutic interventions.

Investing in next-generation sequencing (NGS) technologies is essential. These technologies offer a comprehensive and precise analysis of genetic alterations, facilitating the detection of point mutations, inversions, deletions, and chromosomal rearrangements. Training competent healthcare professionals in genomics and bioinformatics is also crucial to ensure the accurate interpretation of genetic data and the effective implementation of personalized treatments.

By overcoming current obstacles through these investments and developing modern laboratory infrastructures, Morocco can significantly improve the management of cancer patients. Such an approach will not only optimize therapeutic strategies but also increase survival rates and improve patients' quality of life.

## Conclusion

This study represents a major advancement in understanding the molecular profile of lung cancer in Moroccan patients, a field where molecular data was previously largely lacking. Through next-generation sequencing (NGS) technology, we have identified significant genetic alterations, some of which are specific to our cohort while being consistent with global trends. The results obtained enhance our understanding of the genetic alterations present in this specific population. They pave the way for future research aimed at refining diagnostic and therapeutic strategies, thereby improving clinical outcomes for lung cancer patients in Morocco. These findings are essential for the development of personalized treatments and for better clinical management of patients.

### What is known about this topic

- Lung cancer is the leading cause of cancer-related deaths worldwide, with high incidence and mortality rates, particularly among smokers and elderly patients;
- Mutations in the TP53, KRAS, and EGFR genes are frequently found in lung cancer, influencing prognosis and therapeutic options;
- Next-generation sequencing (NGS) technology allows for the rapid and precise identification of genetic mutations, facilitating personalized medicine.

### What this study adds

- This study is the first to use next-generation sequencing to map genetic alterations in Moroccan patients with lung cancer;
- It highlights the genetic diversity of mutations in the Moroccan population, with a high prevalence of mutations in the TP53, KRAS, and EGFR genes, providing novel molecular data for this region;
- The study offers a detailed genomic landscape, including point mutations, gene amplifications, and gene fusions, enabling a better molecular classification of lung cancer subtypes in Morocco.
